# High plasma concentrations of acyl‐coenzyme A binding protein (ACBP) predispose to cardiovascular disease: Evidence for a phylogenetically conserved proaging function of ACBP

**DOI:** 10.1111/acel.13751

**Published:** 2022-12-12

**Authors:** Léa Montégut, Adrien Joseph, Hui Chen, Mahmoud Abdellatif, Christoph Ruckenstuhl, Omar Motiño, Flavia Lambertucci, Gerasimos Anagnostopoulos, Sylvie Lachkar, Silvia Dichtinger, Maria Chiara Maiuri, François Goldwasser, Benoit Blanchet, Frédéric Fumeron, Isabelle Martins, Frank Madeo, Guido Kroemer

**Affiliations:** ^1^ Centre de Recherche des Cordeliers, Equipe labellisée par la Ligue contre le cancer, Inserm U1138 Université Paris Cité, Sorbonne Université Paris France; ^2^ Metabolomics and Cell Biology Platforms Gustave Roussy Institut Villejuif France; ^3^ Faculté de Médecine, Université de Paris Saclay Paris France; ^4^ Service de médecine intensive réanimation Hôpital Saint‐Louis Paris France; ^5^ Department of Cardiology Medical University of Graz Graz Austria; ^6^ BioTechMed‐Graz Graz Austria; ^7^ Institute of Molecular Biosciences, NAWI Graz University of Graz Graz Austria; ^8^ Department of Medical Oncology Cochin Hospital, AP‐HP Paris France; ^9^ URP4466, Université Paris Cité Paris France; ^10^ Pharmacokinetics and Pharmacochemistry Unit Cochin Hospital, Paris Descartes University, CARPEM, AP‐HP Paris France; ^11^ UMR8038 CNRS, U1268 INSERM, Faculty of Pharmacy, University of Paris, PRES Sorbonne Paris Cité, CARPEM Paris France; ^12^ Institut Necker‐Enfants Malades, Université Paris Cité, INSERM UMR‐S1151, CNRS UMR‐S8253 Paris France; ^13^ Field of Excellence BioHealth University of Graz Graz Austria; ^14^ Institut du Cancer Paris CARPEM, Department of Biology Hôpital Européen Georges Pompidou, AP‐HP Paris France

**Keywords:** aging, autophagy, cancer, cardiovascular diseases, diazepam‐binding protein, metabolism

## Abstract

Autophagy defects accelerate aging, while stimulation of autophagy decelerates aging. Acyl‐coenzyme A binding protein (ACBP), which is encoded by a diazepam‐binding inhibitor (DBI), acts as an extracellular feedback regulator of autophagy. As shown here, knockout of the gene coding for the yeast orthologue of ACBP/DBI (*ACB1*) improves chronological aging, and this effect is reversed by knockout of essential autophagy genes (*ATG5*, *ATG7*) but less so by knockout of an essential mitophagy gene (*ATG32*). In humans, ACBP/DBI levels independently correlate with body mass index (BMI) as well as with chronological age. In still‐healthy individuals, we find that high ACBP/DBI levels correlate with future cardiovascular events (such as heart surgery, myocardial infarction, and stroke), an association that is independent of BMI and chronological age, suggesting that ACBP/DBI is indeed a biomarker of “biological” aging. Concurringly, ACBP/DBI plasma concentrations correlate with established cardiovascular risk factors (fasting glucose levels, systolic blood pressure, total free cholesterol, triglycerides), but are inversely correlated with atheroprotective high‐density lipoprotein (HDL). In mice, neutralization of ACBP/DBI through a monoclonal antibody attenuates anthracycline‐induced cardiotoxicity, which is a model of accelerated heart aging. In conclusion, plasma elevation of ACBP/DBI constitutes a novel biomarker of chronological aging and facets of biological aging with a prognostic value in cardiovascular disease.

## Graphical abstract

Montegut et al. provide evidence in favor of a proaging function of acyl CoA binding protein (ACBP) across multiple systems: yeast, mice and humans with cardiovascular disease.

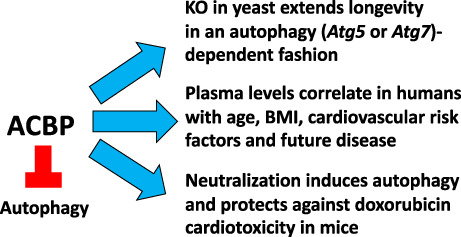

## 1 INTRODUCTION

Acyl‐coenzyme A binding protein (ACBP), which is encoded by a *diazepam‐binding inhibitor* (DBI), is a leaderless polypeptide that can be secreted unconventionally during the activation of autophagy (Bravo‐San Pedro, Sica, Martins, Anagnostopoulos, et al., 2019; Duran et al., 2010; Manjithaya et al., 2010). ACBP/DBI is a phylogenetically conserved protein, the existence of which has been documented in prokaryotes (in particular in eubacteria) as well as in all major eukaryotic kingdoms including animals, fungi, and plants (Du et al., 2016; Thomas et al., 2021). Its autophagy‐dependent release has been reported in unicellular and multicellular fungi (Duran et al., 2010; Manjithaya et al., 2010) as well as in human and murine cells (Bravo‐San Pedro, Sica, Martins, Anagnostopoulos, et al., 2019; Loomis et al., 2010). As an intracellular protein, ACBP/DBI binds to activated medium‐chain fatty acids, facilitating their intracellular transport (Alquier et al., 2021). As an extracellular protein, ACBP/DBI can interact with specific receptors including the GTP‐coupled protein receptor (GCPR) Ste3 (in yeast) (Charmpilas et al., 2020) and the octadecapeptide (ODP) GPCR (in the mouse central nervous system, CNS) (Bouyakdan et al., 2019). Moreover, ACBP/DBI binds to the gamma‐aminobutyric acid (GABA) receptor of the A type (GABA_A_R), a ligand‐gated chloride channel that is expressed by neurons as well as by multiple peripheral (non‐CNS) cell types (Christian et al., 2013; Montégut et al., 2021).

Importantly, the removal of genes encoding ACBP/DBI orthologues from the genomes of *Saccharomyces cerevisiae* (Fabrizio et al., 2010) or *Caenorhabditis elegans* (Shamalnasab et al., 2017) increases lifespan, suggesting that ACBP/DBI is a proaging factor. Indeed, in humans, ACBP/DBI concentrations in the plasma increase with age (Joseph et al., 2021), as well as with BMI (Joseph et al., 2021, 2020), which is a risk factor for accelerated aging (López‐Otín et al., 2016; López‐Otín & Kroemer, 2021). Circulating ACBP/DBI is also increased in patients with Alzheimer's disease (Conti et al., 2021). The mechanisms through which ACBP/DBI might favor the aging process have yet to be addressed mechanistically. However, it appears intriguing that ACBP/DBI acts as an extracellular feedback inhibitor of autophagy (Bravo‐San Pedro, Sica, Martins, Anagnostopoulos, et al., 2019; Bravo‐San Pedro, Sica, Martins, Pol, et al., 2019), knowing that inhibition of autophagy is a major aging accelerator (Klionsky et al., 2021; Rubinsztein et al., 2011).

The mechanisms through which extracellular ACBP/DBI inhibits autophagy have been characterized to some extent. Thus, the depletion of intracellular ACBP/DBI (which results from its secretion) appears to contribute to autophagy inhibition (Bravo‐San Pedro, Sica, Martins, Anagnostopoulos, et al., 2019). The addition of recombinant ACBP/DBI protein to nutrient‐deprived cells or injection of the protein into starved mice reduces autophagy concomitant with the activation of an autophagy‐suppressive signaling pathway involving protein kinase B (PKB, best known as AKT1) and mechanistic target of rapamycin complex‐1 (MTORC1) (Bravo‐San Pedro, Sica, Martins, Anagnostopoulos, et al., 2019). Moreover, ACBP/DBI stimulates feeding behavior through a phylogenetically ancient pathway (Madeo et al., 2020). In turn, caloric intake suppresses autophagy via the provision of nutrients as well as the upregulation of trophic factors including insulin and the insulin‐like growth factor‐1 (IGF1) pathway (Kitada & Koya, 2021; Kroemer et al., 2018; López‐Otín et al., 2016). In mice, a mutation in the gamma‐2 subunit of GABA_A_R that precludes binding of ACBP/DBI (Christian et al., 2013) abolishes appetite stimulation by intravenously injected ACBP/DBI (Joseph et al., 2020).

Intrigued by the aforementioned findings, we decided to investigate the mechanism through which ACBP/DBI depletion enhances longevity in yeast and to address the extent to which human ACBP/DBI might hasten biological aging (as indicated by the precocious manifestation of age‐associated diseases). The results of our study suggest that ACBP/DBI accelerates the aging process via the suppression of autophagy and that ACBP/DBI favors the manifestation of cardiovascular disease in mice and humans.

## 2 RESULTS

### 2.1 Removal of the yeast orthologue of ACBP/DBI enhances longevity through an autophagy‐dependent mechanism

**FIGURE 1:**
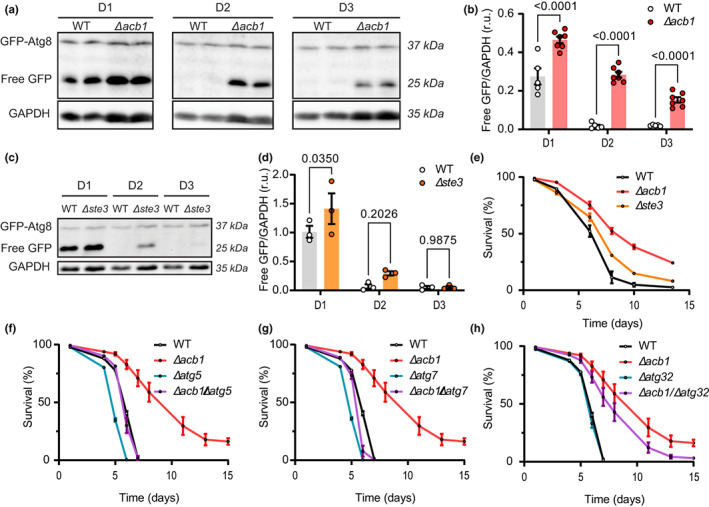
Acb1 (homolog of ACBP) and Ste3—deficient diploid *Saccharomyces cerevisiae* strains show a (macro)autophagy‐dependent increase in chronological lifespan. (a and b) autophagy immunoblotting analysis of protein extracts from wild type (WT) and *∆acb1* cells expressing a chromosomal GFP‐Atg8 fusion protein. Blots were probed with antibodies against GFP to detect GFP‐Atg8 and free GFP, which is indicative of autophagic flux, and against GAPDH as loading control, revealing a significant increase of autophagic flux in *∆acb1* mutant strains. Representative results (a) and densitometric quantification (b) at 1, 2, and 3 days are shown (*n* = 5–7). (c and d) autophagy immunoblotting analysis of protein extracts from wild type (WT) and *∆ste3* (coding for membrane receptor that couples factor a pheromone binding to a MAP kinase cascade) cells expressing a chromosomal GFP‐Atg8 fusion protein. Blots were probed with antibodies against GFP to detect GFP‐Atg8 and free GFP, which is indicative of autophagic flux, and against GAPDH as the loading control. Representative results (c) and densitometric quantification (d) at 1, 2, and 3 days are shown (*n* = 3). Quantitative results are reported as means ± SEM. (e) Chronological aging experiments of wild type and *STE3* and *ACB1* single gene deletion mutants (∆*acb1* and ∆*ste3*) (*n* = 4; *p* values obtained by 2‐way ANOVA: Wt vs. ∆*acb1 p* = 5 E‐10; wt vs. ∆*ste3 p* = 0.0002; ∆*acb1* vs. ∆*ste3 p* < 1 E‐11). (f–h) Chronological aging experiments of *ACB1* and autophagy‐incompetent *ATG* single gene deletion mutants (∆*acb1*, ∆*atg5* (f), ∆*atg7* (g), and ∆*atg32* (h)) as well as double‐mutants thereof (∆*acb1*/∆*atg5, ∆acb1/∆atg7* and ∆*acb1*/∆*atg32*). Prolonged CLS by Acb1 deficiency depended on the macro‐autophagic core machinery (Atg5 and Atg7) but was largely independent of mitophagy (Atg32). Strains were grown in batch cultures, dead cells were identified via flow cytometry analysis following propidium iodide (pi) staining and survival was normalized to day one (*n* = 4 to 6; *p* values obtained by 2‐way ANOVA: Wt vs ∆*acb1 p* = 7.9 E‐08; wt vs ∆*atg5 p* = 1.3 E‐08; wt vs ∆*acb1*/*atg5 p* = 0.766; ∆*acb1* vs ∆*acb1*/*atg5 p* = 1.4 E‐08; ∆*atg5* vs ∆*acb1*/*atg5 p* = 2.5 E‐09; wt vs ∆*atg7 p* = 1.5 E‐08; wt vs ∆*acb1*/*atg7 p* = 0.004; ∆*acb1* vs ∆*acb1*/*atg7 p* = 2 E‐10; ∆*atg7* vs ∆*acb1*/*atg7 p* = 8.7 E‐07; wt vs ∆*atg32 p* = 0.082; wt vs ∆*acb1*/*atg32 p* = 3.5 E‐07; ∆*acb1* vs ∆*acb1*/*atg32 p* = 1.8 E‐05; ∆*atg32* vs ∆*acb1*/*atg32 p* = 1.7 E‐09). Statistical comparisons were performed by 2‐way ANOVA (b, d–h) and the corresponding *p* values are reported on the plots or in their legend.

Diploid *Saccharomyces cerevisiae* (yeast) cells subjected to the knockout of the yeast gene *ACB1* (which encodes the ACBP/DBI orthologue) exhibit an increase in autophagic flux, as indicated by an increase in free GFP generated from a GFP‐Atg8 fusion protein compared to wild type (WT) controls (Figure 1a,b). An increase in autophagic flux was also observed in yeast cells lacking *STE3* (which codes for the yeast ACBP/DBI receptor) (Figure 1c,d). In chronological survival experiments, *Δacb1* and *Δste3* cells both exhibited improved longevity compared to WT controls (Figure 1e). Moreover, simultaneous knockout of the essential autophagy genes *ATG5* and *ATG7* largely abolished the longevity‐extending effects of *Δacb1* (Figure 1f,g). In contrast, knockout of the specific mitophagy gene *ATG32* had a less dramatic (but still significant) negative effect on the antiaging effect of *Δacb1* (Figure 1h). We also noted that *Δacb1* cells were more resistant to cell death (propidium iodine positivity) by heat stress (50°C for 15 min) than WT cells (Figure S1A). However, this effect was not reverted by simultaneous removal of *ATG5*, *ATG7*, or *ATG32* (Figure S1B).

In sum, removal of the yeast ACBP/DBI orthologue has longevity‐extending and cytoprotective effects, which depend at least in part on autophagy.

### 2.2 High ACBP/DBI plasma levels predict future disease in an exploration cohort

**FIGURE 2:**
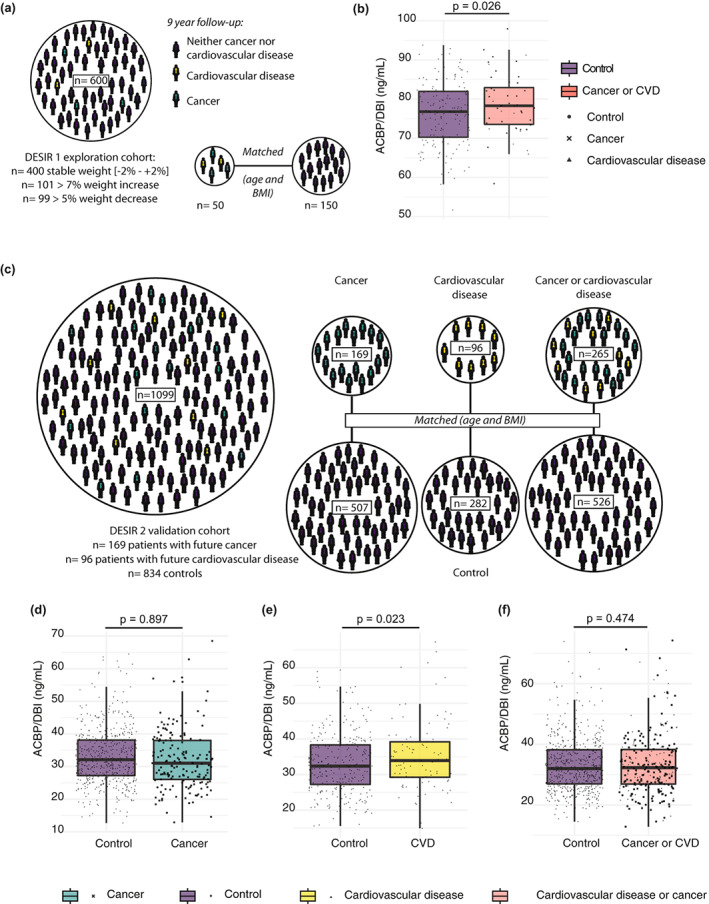
Patients who will develop age‐related pathologies have increased ACBP/DBI levels in the exploration cohort (DESIR 1) and the validation cohort (DESIR 2). (a) The exploration cohort was derived from a weight gain and loss cohort (DESIR 1), of which were drawn the patients who developed cancer or cardiovascular disease during the 9 years follow‐up period and controls matched by age and BMI. (b) Comparison of ACBP/DBI plasma levels (ng/ml) from patients with future cancer or cardiovascular disease versus control individuals from the exploration cohort shows increased levels in patients with future age‐associated diseases. Statistical comparison was performed by one‐sided Student's *t*‐test and the corresponding p‐value is reported. (c) The validation cohort (DESIR 2) was selected with the same method as the exploration cohort but among the whole DESIR study patients (*n* = 5212), except for the ones already included in DESIR 1. The final number of patients included those who developed cancer or cardiovascular disease (*n* = 265) and healthy controls (*n* = 834). ACBP/DBI plasma levels (ng/ml) of the DESIR 2 patients with the future diagnosis of cancer (d), cardiovascular disease (e), or both (f) as well as their respective controls. Control patients are those who developed neither of these two pathologies during the 9‐years follow‐up period, matched by age and body mass index (BMI) to each case (3:1, respectively). Statistical comparisons were performed by one‐sided Student's *t*‐test and the corresponding *p*‐value is reported on the boxplots.

We previously investigated the effects of different BMI trajectories (increase, stability, or decrease) on ACBP/DBI concentrations within the DESIR cohort (Joseph et al., 2021). In this selected DESIR subpopulation of 600 relatively healthy individuals (with or without prediabetes) (Table S1), 50 developed cardiovascular events (such as heart surgery, myocardial infarction, and stroke) and/or developed cancer within a time window of 9 years after blood sampling. However, these patients were also older and, at least in the case of CVD, had a higher BMI (Table S1). Therefore, we matched every single patient among the 50 “cases” with 3 “controls” who had similar BMI and age but did not develop CVD or cancer (Figure 2a). Of note, in this exploration cohort, the 50 cases did exhibit significantly higher ACBP/DBI levels (*p* = 0.026, Student's *t*‐test) than the 150 matched controls (Figure 2b). As a result, we hypothesized that such individuals developing CVD or cancer might be biologically more “aged” than individuals of a similar chronological age or BMI and hence exhibit relatively higher ACBP/DBI levels as a biomarker of biological aging. To validate this notion, we decided to generate an in‐house ELISA to quantify human ACBP/DBI levels at a reduced cost (Figure S2) in a larger “validation” cohort, composed of the remaining probands of the DESIR cohort.

### 2.3 High circulating ACBP/DBI levels predict future cardiovascular events in the validation cohort

Our independent validation cohort (*n* = 1099) consisted of all other patients within the DESIR cohort who developed cancer (*n* = 169) or CVD (*n* = 96) compared to randomly selected cancer‐ and CVD‐free controls (*n* = 834) (Figure 2c; Table S2). Interestingly, ACBP/DBI levels were elevated only in patients developing CVD (Figure S3A). However, the mean age and BMI were expectedly higher in these individuals than in controls (Figure S3B,C). To investigate whether the association between CVD and high ACBP/DBI levels was independent of chronological age and BMI, we matched each case with 2 to 3 controls with similar age and BMI (Figure 2c). This stratified analysis revealed again that CVD (but not cancer nor the aggregate of CVD plus cancer) is associated with a statistically significant (*p* = 0.023, Student's *t*‐test) increase in the level of ACBP/DBI (Figure 2d–f), even at a comparable age and BMI (Figure S4).

In conclusion, high plasma concentrations of ACBP/DBI are associated with an elevated risk of future CVD.

### 2.4 Future or present cardiovascular or malignant disease weakens the correlation between ACBP/DBI levels and BMI

**FIGURE 3:**
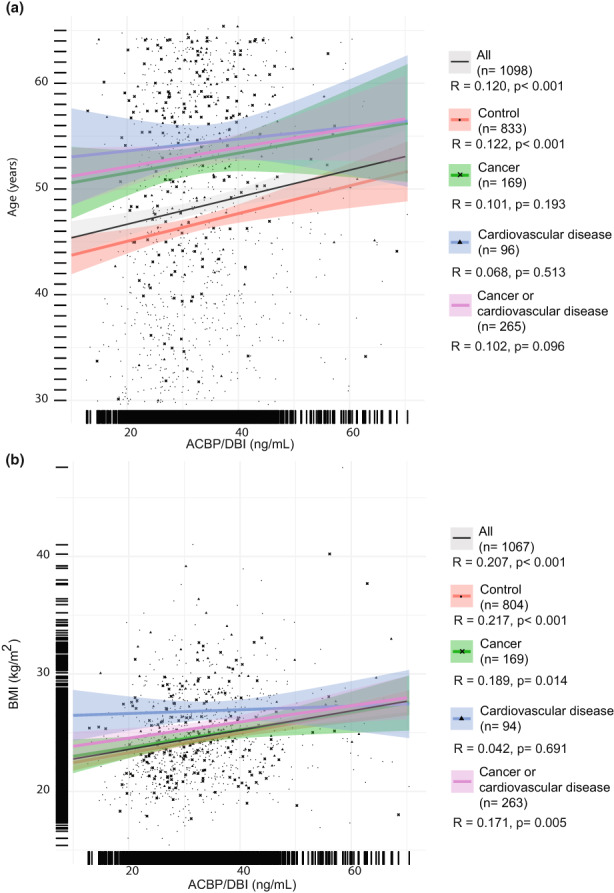
The cardiovascular disease‐related increase in ACBP is above that induced by age or body mass index (BMI). The expected positive correlations between ACBP/DBI and age (a), or BMI (b) are observed in the DESIR 2 cohort at the whole‐cohort level (*n* = 1098) and in controls who developed neither of the two analyzed age‐related pathologies (*n* = 833). The correlation with age (a) is not significant in the categories of patients who developed cancer (*n* = 169), cardiovascular disease (*n* = 96), or one of these two complications (*n* = 265). The correlation with BMI (b) is maintained in all groups of patients except for the ones who developed cardiovascular disease (*n* = 94). Pearson's correlation coefficient (*R*) with their *p*‐value and the number of samples available (n) are shown in the legend of each panel.

**FIGURE 4:**
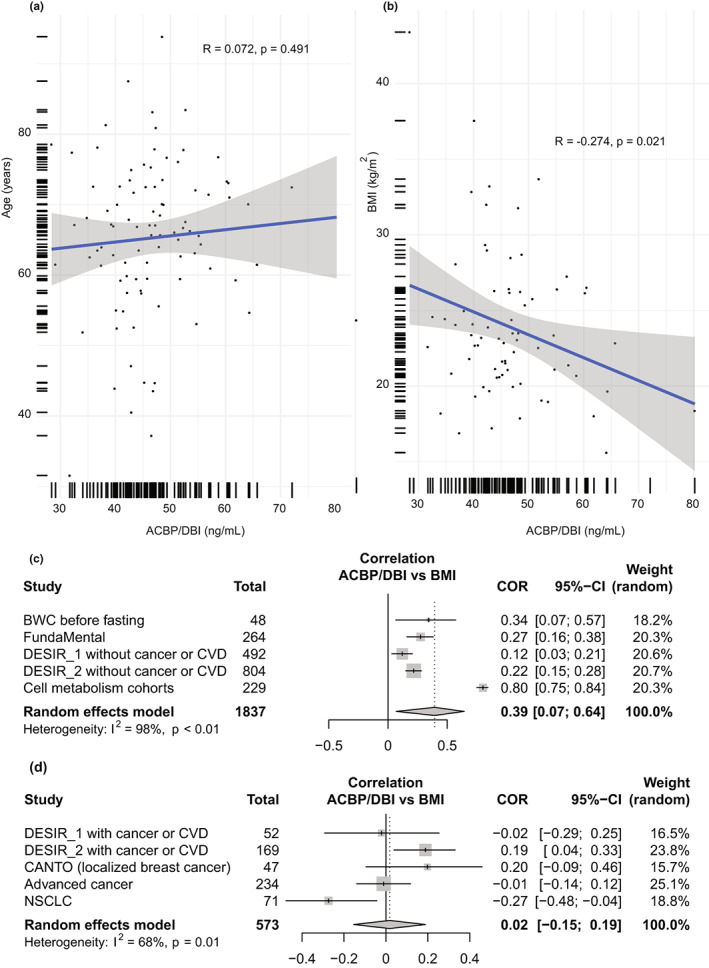
A meta‐analysis of publicly available cohorts confirms the weakened correlation between ACBP/DBI plasma levels and age or body mass index (BMI) in the presence of age‐related pathologies. ACBP/DBI levels in the plasma (ng/ml) of patients diagnosed with stage 3 or stage 4 nonsmall cell lung cancer from Cochin hospital were compared by scatter plot with linear regression line with their age (a) or and body mass index (kg/m^2^) (b). Pearson's correlation coefficient (*R*) and the associated p values are presented in the legend of each panel, showing a weakening of the correlation of ACBP/DBI with age and a reversion of this correlation to an anticorrelation with BMI. The meta‐analysis of all publicly available cohorts was performed to discriminate between people with cancer or cardiovascular disease (CVD), as well as in patients with neither of these pathologies. Each Pearson's correlation coefficient (COR) is represented with its 95% confidence interval. The size of the square around the COR value is proportional to the sample size of the study. The pooled correlation coefficient was calculated using a random effect model and presented with its 95% confidence interval.

Across the entire DESIR 2 cohort, ACBP/DBI levels correlated with age (Figure 3a) and BMI (Figure 3b). Such correlations between ACBP/DBI and age or BMI persisted in control subjects but were lost in patients with cardiovascular disease (both for age and BMI; Figure 3a,b) or cancer (for age only; Figure 3a), in whom ACBP was elevated even at a young age and low BMI. To confirm this finding in the context of extensive pathological aging, we measured ACBP/DBI plasma levels in another cohort of patients with advanced cancer. Patients that had been diagnosed with stage 3 or 4 nonsmall cell lung cancer (NSCLC) also lost the correlation of ACBP/DBI with age (Figure 4a) and even manifested a reverse correlation of ACBP/DBI with BMI (Figure 4b), perhaps due to the advanced stage of cancer in this cohort. Regardless, a meta‐analysis of publicly available data confirmed that the association of ACBP/DBI with BMI was well seen in patients without major diseases across different cohorts (*r* = 0.37; 95% CI = 0.07–0.64) (Figure 4c) but was lost for patients with current or future diagnosis of CVD or cancer (*r* = 0.02; 95% CI = −0.15–0.19) (Figure 4d). In contrast, the correlation of ACBP/DBI with chronological age was conserved across different cohorts (*r* = 0.21; 95% CI = 0.06–0.34), even in the presence (or future development) of CVD or malignant disease, albeit to a lower extent (*r* = 0.14; 95% CI = 0.05–0.23) (Figure S6).

In conclusion, it appears that the statistical relationship between circulating ACBP/DBI concentrations and BMI is weakened in individuals that have or will develop CVD or cancer.

### 2.5 ACBP/DBI concentrations correlate with cardiovascular risk factors

**FIGURE 5:**
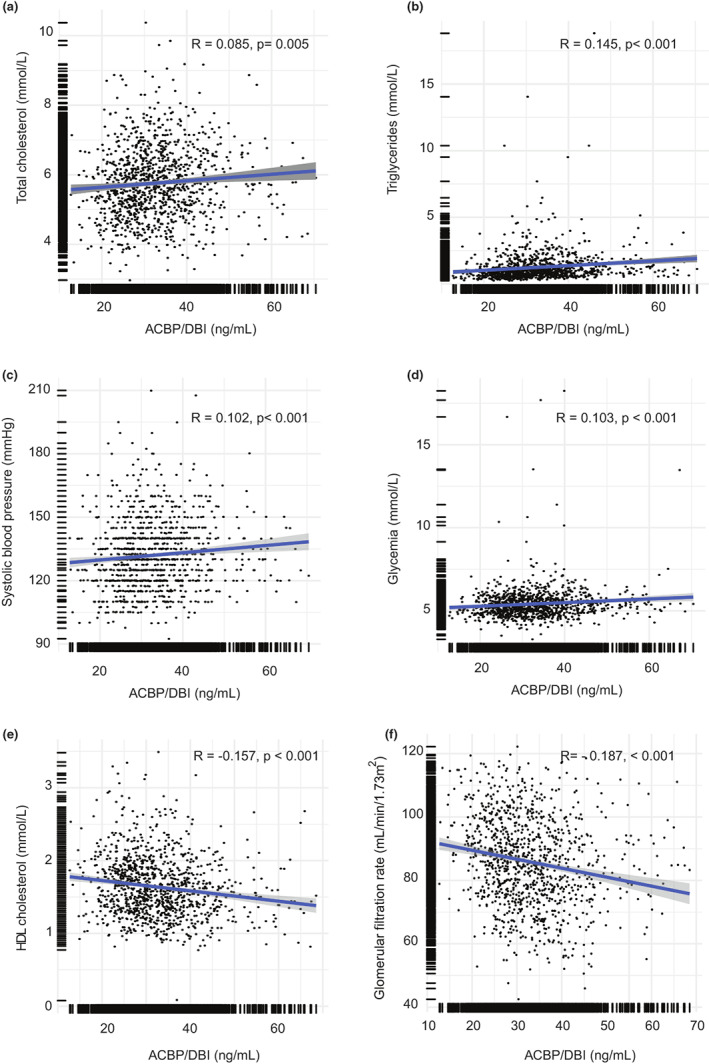
ACBP/DBI correlates with conventional cardiovascular risk factors.The correlation of ACBP/DBI with clinical blood parameters was performed in all patients from DESIR 2. Total cholesterol (a), triglycerides (b), systolic blood pressure (c), glycemia (d) correlate positively with the plasma concentration of ACBP/DBI, while and HDL (e) and glomerular filtration rate (f) are inversely correlated with plasma ACBP/DBI. Statistical analysis was performed by linear regression and Pearson's correlation coefficient (*R*) with the associated *p*‐value and the number of samples available (*n*) are shown in the legend of each panel.

As to be expected, patients with future CVD manifested CVD‐associated risk factors including higher fasting glucose levels, total cholesterol, triglycerides, and systolic blood pressure but lower high‐density lipoprotein (HDL) levels (Figure S8). Therefore, we determined the relationship between ACBP/DBI concentrations and cardiometabolic risk factors. Of note, ACBP/DBI positively correlated with total cholesterol levels (Figure 5a), triglycerides (Figure 5b), systolic blood pressure (Figure 5c), and glucose levels (Figure 5d), but inversely correlated with HDL cholesterol (Figure 5e) and glomerular filtration rate (Figure 5f). Among these associations, the correlations between ACBP/DBI and triglycerides (*p* = 0.034) and HDL cholesterol (*p* < 0.001) were independent of age and BMI in a multivariate regression model. Beyond these associations, high ACBP/DBI concentrations also correlated with high creatinine levels and reduced glomerular filtration, confirming our previous observation that elevated ACBP/DBI is associated with renal dysfunction (Joseph et al., 2021).

In sum, ACBP/DBI plasma concentrations correlate with known cardiovascular risk factors.

### 2.6 ACBP/DBI neutralization reduces anthracycline‐accelerated cardiac aging

**FIGURE 6:**
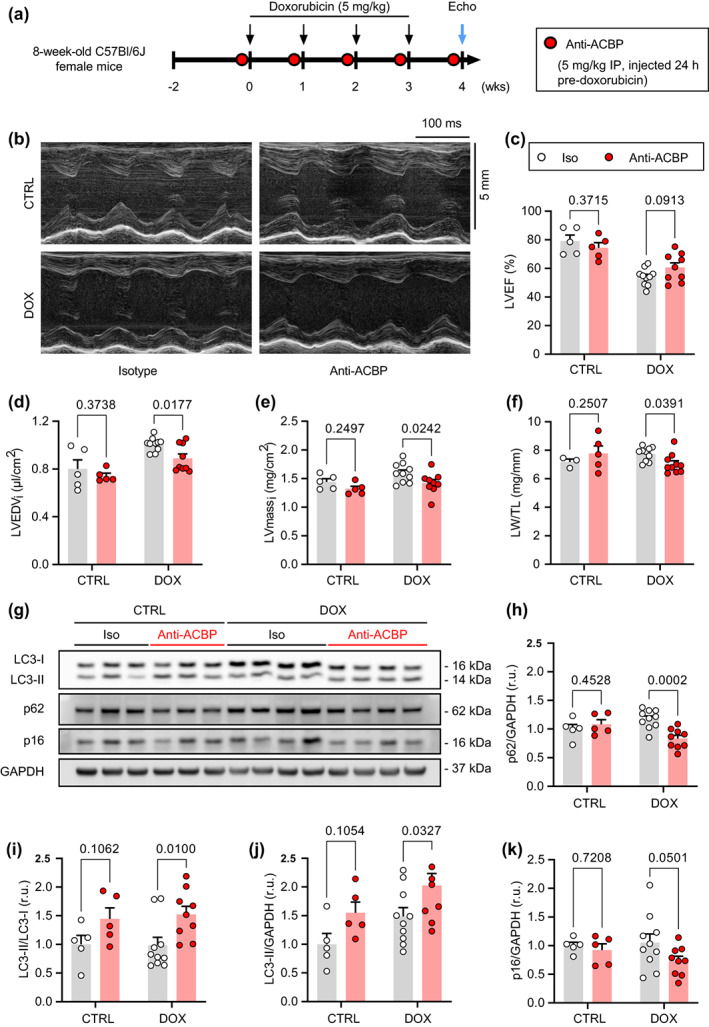
ACBP neutralization attenuates anthracyline‐induced cardiotoxicity. (a) Doxorubicin (DOX) was administered to C57Bl/6J female mice, which were also treated with an ACBP‐neutralizing antibody (anti‐ACBP) or mouse isotype IgG (CTRL) for the indicated period, before undergoing echocardiography‐based assessment of the heart. (b) Representative echocardiography‐derived left ventricular (LV) M‐mode tracings. (c) LV ejection fraction (LVEF). (d) LV end‐diastolic volume normalized to body surface area (LVEDV_i_). (e) LV mass index (LVmass_i_), calculated as the ratio between LVmass and body surface area. (f) Tibia length‐normalized lung weight (LW/TL) (g) representative immunoblot of hearts (one heart per lane) from mice of the four treatment groups. (h–k) quantification and statistical analyses of immunoblots from all mice included in the experiment. *N* = 3–10 mice per group. *p* values in (c–f, h–k) represent pairwise comparisons between anti‐ACBP‐treated mice and their respective isotype (iso)‐treated controls using simple main effects of a factorial ANOVA. Bars and error bars show means and SEM, respectively, with individual data points superimposed. Echo, Echocardiography; GAPDH, glyceraldehyde‐3‐phosphate dehydrogenase; LC3, Microtubule‐associated proteins 1A/1B light chain 3B; p16, Cyclin‐dependent kinase inhibitor 2A (CDKN2A); p62, Sequestosome‐1 (SQSTM1).

We used an animal model of anthracycline‐induced cardiomyocyte senescence (Maejima et al., 2008; Piegari et al., 2013) to determine whether ACBP is causally involved in cardiac disease (Figure 6a). Mice were subjected to chronic doxorubicin (DOX) treatment (cumulative dose: 20 mg/kg body weight, injected intraperitoneally (*i.p*.) over 4 weeks). DOX treatment reduced left ventricular ejection fraction (*p* < 0.001, Figure 6b,c), thereby causing ventricular dilation (*p* = 0.001; Figure 6d). By contrast, treatment of DOX mice with a murine monoclonal ACBP‐neutralizing antibody (anti‐ACBP; 5 mg/kg body weight, injected *i.p*. weekly) partially preserved cardiac function, as suggested by a significant reduction in left ventricular dilation, despite an unaltered ejection fraction (Figure 6c,d). Anti‐ACBP‐treated mice also exhibited lower left ventricular mass index and tibia length‐normalized lung weight (Figure 6e,f), indicating reduced cardiac remodeling and lung congestion, respectively. Of note, doxorubicin‐induced suppression of body weight gain was not affected by anti‐ACBP (Figure S9A), whereas anti‐ACBP appeared to increase heart rate, irrespective of DOX treatment (Figure S9B).

Immunoblot analyses (Figure 6g) revealed that ACBP neutralization induced autophagic flux in the hearts of DOX‐treated mice, as indicated by a reduction in the abundance of the autophagic substrate sequestosome‐1 (SQSTM1, best known as p62) (Figure 6h) and an increased ratio of the lipidated, autophagy‐associated form (II) of microtubule‐associated proteins 1A/1B light chain 3B (best known as LC3) over its nonlipidated form (I) (Figure 6i) or the loading control glyceraldehyde‐3‐phosphate dehydrogenase (GAPDH) (Figure 6j). Moreover, the abundance of the senescence marker cyclin‐dependent kinase inhibitor 2A (CDKN2A, best known as p16), which has been causatively involved in DOX‐induced cardiac failure (Demaria et al., 2017), was reduced by ACBP neutralization (Figure 6k).

Altogether these results indicate that ACBP neutralization reduces the cardiotoxicity of elevated anthracycline doses.

## 3 DISCUSSION

Autophagy is the most efficient mechanism for turning over cytoplasmic organelles or other higher‐order structures, such as protein aggregates, that cannot be digested by cytosolic enzymes or proteasomes (Deretic & Kroemer, 2021; Klionsky et al., 2021). As such, autophagy plays an essential role in avoiding the accumulation of unwarranted waste material and dysfunctional organelles, hence reducing the pace of aging (Hansen et al., 2018; Kitada & Koya, 2021). This antiaging function of autophagy is further reinforced by the fact that malfunctioning organelles such as depolarized mitochondria are selectively marked for preferential destruction by the autophagic machinery, hence contributing to organellar quality control (Green et al., 2011; Longhi et al., 1988).

Given these premises, it is not surprising that genetic, pharmacological, or nutritional interventions designed to enhance autophagy prolong healthspan and lifespan in model organisms including mice. Such strategies include transgenic overexpression of ATG5 (Pyo et al., 2013), gain‐of‐function knock‐in mutation of Beclin 1 (Fernández et al., 2018), administration of the pharmacological autophagy inducers rapamycin (Harrison et al., 2009) and spermidine (Eisenberg et al., 2016), as well as gross nutritional interventions such as caloric restriction (Mitchell et al., 2019), intermittent fasting (Mattson et al., 2017), selective reduction of carbohydrates (Kroemer et al., 2018) or administration of ketone bodies (Asadi Shahmirzadi et al., 2020). Among these autophagy inducers, spermidine has been specifically analyzed for its effects on normal cardiac aging and has been found to inhibit cardiac failure through a mechanism requiring autophagy induction in cardiomyocytes (Eisenberg et al., 2016). We found that ACBP/DBI neutralization triggers autophagy in the heart muscle and reduces doxorubicin‐induced heart damage, which is considered a model of accelerated heart aging (Demaria et al., 2017). However, it remains to be demonstrated that ACBP/DBI inhibition can retard normal cardiac aging as well. Of note, old age is linked to a progressive decline in autophagic turnover (Hansen et al., 2018; Kitada & Koya, 2021), which may contribute to the progressive deterioration of organellar, cellular, and organ functions (López‐Otín & Kroemer, 2021).

In this vein, it appears logical that the removal of ACBP/DBI, which is an extracellular feedback inhibitor of autophagy, can extend the longevity of yeast in an autophagy‐dependent fashion. This antiaging pathway appears to be phylogenetically ancient, because knockout of ACBP3 (which is one of the plant ACBP/DBI orthologues), delays leaf senescence in *Arabidopsis thaliana*, while its overexpression accelerates senescence and disrupts autophagosome formation (Xiao et al., 2010). The leaf chlorosis‐inducing effect of ACBP has been confirmed for another plant species, *Brassica napus* (Ling et al., 2018). In *C. elegans*, the removal of one ACBP orthologue, ACBP1 (also called *maa‐1*) promotes lifespan extension and resistance to different types of stress (Charmpilas et al., 2020). However, it remains to be determined whether this effect is mediated by enhanced autophagy. Moreover, the long‐term effects of ACBP/DBI neutralization on aging in mammals should be determined beyond its positive effect on whole‐body metabolism including the avoidance of weight gain and type‐2 diabetes (Bravo‐San Pedro, Sica, Martins, Anagnostopoulos, et al., 2019).

If ACBP/DBI is a proaging factor, it appears logical that, in humans, ACBP/DBI plasma levels tend to increase with chronological aging (measured in years) and more so with biological aging (indicated by the imminent development of CVD). That said, the source of circulating ACBP/DBI levels remains to be determined. Indeed, it is unclear, which aging tissues and cell types shed ACBP/DBI. Moreover, considering that aging is associated with an inhibition of autophagy (Hansen et al., 2018; Kitada & Koya, 2021), the mechanism through which ACBP/DBI is secreted during aging remains elusive. The senescence‐associated secretory phenotype (SASP) is characterized by the enhanced unconventional secretion of leaderless proteins including interleukins‐1 and 33 as well as HMGB1 (Daniels & Brough, 2017). However, we are not aware of any report describing the senescence‐associated secretion of ACBP/DBI. In obesity, another condition in which autophagy is suppressed (Kitada & Koya, 2021; López‐Otín et al., 2016), circulating ACBP/DBI levels are elevated, correlating with increased mRNA and protein levels of ACBP/DBI in the liver (in mice) and in adipose tissues (in mice and humans) (Bravo‐San Pedro, Sica, Martins, Anagnostopoulos, et al., 2019), suggesting that transcriptional upregulation accounts for the surge in circulating ACBP/DBI protein (Anagnostopoulos et al., 2022). Indeed, ACBP/DBI mRNA has been found upregulated in the liver of aged mice (Wang et al., 2021), as well as in the brain of aged *Macaca fascicularis* (https://ngdc.cncb.ac.cn), suggesting that an age‐associated transcriptional upregulation might contribute to the elevation of plasma ACBP/DBI. However, this hypothesis requires further in‐depth scrutiny. Moreover, the possibility that ACBP/DBI neutralization reduces cellular senescence (as this is suggested by the reversal of doxorubicin‐induced CDKN2A protein levels in the heart) requires further confirmation by investigating the precise cell types that are affected and by monitoring additional senescence markers.

Here, we report that ACBP/DBI plasma levels increased before the development of CVD. This association appears to be independent of chronological age and BMI despite the low number of patients included in this study (*n* = 96). ACBP/DBI is also independently associated with several cardiometabolic risk factors including dyslipidemia (with an increase of total cholesterol and triglycerides but a decrease of HDL) and high systolic blood pressure. These results suggest a causal implication of ACBP/DBI in cardiovascular aging, commensurate with the well‐established atherosclerosis‐preventive and cardioprotective effects of autophagy (Abdellatif et al., 2018; Heusch, 2020; Sciarretta et al., 2021). Indeed, the neutralization of ACBP/DBI protects the heart against ischemic damage in an autophagy‐dependent fashion (Motiño et al., 2022). Moreover, in a cohort of patients infected by the human immunodeficiency virus, plasma ACBP concentrations correlated with those of interleukin‐1β (Isnard et al., 2022), which is a major accelerator of human atherosclerosis (Ridker et al., 2017). Indeed, in mice, ACBP/DBI positively regulates the transcription of the genes coding for interleukin‐1β itself as well as for the interleukin‐1β activator NLRP3 (Motiño et al., 2022.). Future studies must address the precise molecular mechanisms through which ACBP/DBI neutralization may prevent CVD in preclinical models and explore the clinical applicability of these findings. It remains to be seen whether elevated plasma levels of ACBP/DBI may be combined with other biomarkers (e.g., of inflammation) to yield a composite “clock” informing on the actual biological age of an individual. Moreover, the possibility should be explored to combine ACBP/DBI neutralization with additional anti‐inflammatory agents for the prevention of CVD and other age‐associated diseases.

## 4 MATERIAL AND METHODS

### 4.1 Yeast experiments

Strains. Wild type diploid *S. cerevisiae* strain BY4743 (MATa/α *his3Δ1 leu2Δ0 MET15/met15Δ0 LYS2/lys2Δ0 ura3Δ0*) and the homozygous deletion strain BY4743 Δ*acb1* (Δ*acb1*::*URA3*/Δ*acb1*::*URA3*) or Δ*acb1*::*HIS3*/Δ*acb1*::*HIS3* as well as BY4743 Δ*ste3* (Δ*ste3*::*HIS3*/Δ*ste3*::*LEU3*) were used. Macroautophagy‐incompetent BY4743 strains were used as single deletions (Δ*atg5*::*URA3*/Δ*atg5*::*URA3 and* Δ*atg7*::*URA3*/Δ*atg7*::*URA3*) or as double deletion mutants in combination with BY4743 Δ*acb1*. Analyses of autophagy in yeast were carried out in respective BY4743 p8 (GFP‐ATG8) strains as described elsewhere (Eisenberg et al., 2014). Deletions of *ACB1* were made in the haploid variants BY4741 (MATa *his3Δ1 leu2Δ0 met15Δ0 ura3Δ0*) and BY4742 (MATα *his3Δ1 leu2Δ0 lys2Δ0 ura3Δ0*), respectively, following standard protocols with PCR generated cassettes using primers ACB1_fw and ACB1_rev and pUG72 (URA3 marker) as described previously (Charmpilas et al., 2020). For autophagy experiments, the haploid variants BY4741 GFP‐Atg8 and BY4742 GFP‐Atg8, carrying chromosomally N‐terminal tagged ATG8 with GFP, were used with HIS3 marker cassettes (pUG6) (Gueldener, 2002; Guldener, 1996). Transformation was done using the lithium acetate method (Gietz et al., 1995). Deletion was verified by PCR (Primer ACB1_ctrl) (Charmpilas et al., 2020) and the corresponding control Primer (Gueldener, 2002; Sheff & Thorn, 2004). The generated haploid knockout strains were mated and selected for diploids on media devoid of methionine and lysine. Deletion of STE3 (generating sterile strains) was made in the diploid variants BY4743 and BY4743 GFP‐Atg8/GFP‐Atg8 with LEU2 and HIS3 marker cassettes as described above with primers STE3_fw (5′‐AGGCAATTAAATTTGTGTAGGAAAGGCAAAATACTATCAAAATTTTCcagctgaagcttcgtacgc‐3′), STE3_rev (5′‐AAAATAAAATACTCCTAGTCCAGTAAATATAATGCGACACTCTTGTGgcataggccactagtggatc‐3′), and STE3_ctrl (5′‐GTACCACATTGCCAGATTTATGA‐3′).

#### 4.1.1 Chronological aging and heat stress experiments

Yeast strains were inoculated when they reached an OD_600nm_ 0.05 from fresh overnight cultures and grown at 28°C on synthetic minimal medium containing 0.17% yeast nitrogen base (Difco), 0.5% (NH_4_)_2_SO_4_ and 30 mg/L of all amino acids (except for 80 mg/L histidine, and 200 mg/L leucine), 30 mg/L adenine and 320 mg/L uracil with 2% glucose as previously described (Ruckenstuhl et al., 2014). For heat stress experiments cells from the mid‐log phase were subjected to 50°C for 15 min or left at 28°C as a control. To determine CLS, samples were harvested at indicated time points, stained with Propidium iodide (Pi) and Pi‐positive cells were identified via FACS analysis. The %‐Survival was calculated from the unstained population, normalized to survival on day one.

#### 4.1.2 Western‐blot analysis of autophagy

Autophagy was analyzed using cells with a GFP‐Atg8 fusion protein as previously described (Eisenberg et al., 2009). To monitor GFP liberation indicative of an autophagic vacuolar breakdown of GFP‐Atg8, equal amounts of cells were harvested at given time points and subjected to chemical lysis followed by SDS‐PAGE and western blot using standard protocols (Madeo et al., 2002). Blots were probed with anti‐GFP (Roche, #11814460001) and antiglyceraldehyde 3‐phosphate dehydrogenase (GAPDH) Loading Control Monoclonal Antibody (GA1R from Invitrogen, #MA5‐15738) and the respective peroxidase‐conjugated secondary antibodies (Sigma). Densitometric quantification of immunoblots was performed with Image Lab 5.2 Software (Bio‐Rad), and the ratio Free GFP/GAPDH or Acb1/GAPDH was plotted.

### 4.2 Human plasma ACBI/DBI measurements in the DESIR and NSCLC cohorts

Plasma ACBP/DBI levels were measured using an in‐house ELISA as previously described (Montégut et al., 2022), optimized for human use as illustrated in Figure S2. Briefly, high‐binding 96‐well plates (Corning, #9018) were coated with antihuman ACBP/DBI (MyBioScience, #MBS768488) by incubating them in phosphate‐buffered saline containing 0.5 μg/ml of capture antibody for 15 h at 4°C. After blocking, the plasma samples were incubated for 2 h at 20–24°C and detected with a 1 μg/ml solution of biotin‐conjugated capture antibody directed against human ACBP/DBI (MyBioScience, #MBS2003225), also incubated for 2 h at 20–24°C. For colorimetric assessment, a 1:1000 solution of horseradish peroxidase (HRP) enzyme coupled with avidin (BioLegend, #405103) was incubated for 30 min at 20–24°C. Finally, an enzymatic reaction was performed by adding 100 μl of TMB substrate (Thermo Fisher Scientific, # 34028) to each well and stopped by the addition of 50 μl of 2 M sulfuric acid. Extensive rinsing was performed between each step using a Tris‐Buffered Saline 1X solution (Euromedex, # ET220‐B) containing 0.05% Tween 20 (Euromedex, # 2001‐C). ACBP/DBI measurements above or below the standard range were excluded.

The DESIR (*Données Épidémiologiques sur le Syndrome d'Insulino‐Résistance*) study (Balkau et al., 2008) is a 9‐year prospective cohort including 5212 volunteers from the general population at 10 health examination centers in western France. Biological samples were taken, and BMI was measured at inclusion, then every 3 years for 9 years. In this study, we measured the plasma ACBP/DBI levels at inclusion and compared them to clinical data from the whole follow‐up.

We first tested our hypothesis in an exploratory cohort including individuals who gained (+7%, *n* = 101) or lost weight (−5%, *n* = 99) during their 9‐year follow‐up and compared each of them to 2 control individuals whose weight remained stable [between −2% and +2%, *n* = 394]. ACBP/DBI measurements from these individuals in relation to their weight evolution have been published elsewhere (Joseph et al., 2021). We identified patients from this cohort who developed an age‐related disease (cancer or cardiovascular disease, *n* = 50) later during their follow‐up and compared their baseline ACBP/DBI levels with controls matched for age and BMI.

To confirm our hypothesis, we used a validation cohort in which we included all patients from the DESIR cohort who developed cancer or cardiovascular disease during their 9‐year follow‐up (*n* = 265). Three random controls who developed neither of these diseases were included for each case. We performed a planned nested case–control analysis in which patients were matched with controls with similar age and BMI.

The protocol for this study was registered with Open Science Foundation on 2021‐04‐29.

We also measured ACBP/DBI in the plasma of nonsmall cell lung cancer patients (*n* = 71, NCT04879316) after their first‐line systemic therapy from Cochin Hospital, to validate our previous findings (Joseph et al., 2021) that the correlation between ACBP/DBI and BMI was lost in cancer patients.

### 4.3 Mouse experiments

#### 4.3.1 Animal housing and treatment

8‐week‐old C57Bl/6J female mice were purchased from Envigo (Gannat, France). Following one week of acclimatization, mice were randomized (1:1) to receive either mouse monoclonal anti‐ACBP‐neutralizing antibody (5 mg/kg body weight, injected *i.p*. once per week, Fred Hutch Antibody Technology clone 7a) or its isotype IgG2a (negative control; 5 mg/kg body weight, BioXCell, clone 2A3, #BE0089). Each group was further randomized (1:2) to receive a weekly intraperitoneal injection of saline or doxorubicin (DOX, 5 mg/kg body weight; a cumulative dose of 20 mg/kg body weight over 4 weeks, Sigma, #MKCM8540) (Li et al., 2018). Of note, anti‐ACBP was always administered 24 hours prior to doxorubicin injection.

All mice used in this study were housed in a temperature‐controlled environment with 12 h light/dark cycles and ad libitum access to water and food (standard chow diet; #A04, Safe). Animal experiments were performed according to the European ethical regulations (Directive 2010/63/EU) and were approved by the responsible Animal Experimental Ethics Committee (protocol #35132–202202021617318).

#### 4.3.2 Ultrasound assessment of cardiac function

Mice were assessed using noninvasive echocardiography (Vevo3100, Fujifilm VisualSonics Inc.) the week following the last dose of DOX. Briefly, mice were minimally anesthetized (4%–5% isoflurane for induction; 0.5% for maintenance), and body temperature was kept at 37°C using a temperature‐controlled heating platform. Mice were placed in a supine position with their limbs in direct contact with noninvasive electrocardiogram leads for heart rate assessment. Prewarmed ultrasound transmission gel was spread on the shaved chest to obtain cardiac tracings in the parasternal long axis using a high‐resolution 55 MHz linear‐array probe. M‐mode tracings were used to evaluate cardiac wall thickness and internal left ventricular dimensions at the level of the papillary muscles during systole and diastole, as previously described (Abdellatif et al., 2016, 2022). Ventricular volumes and myocardial mass were estimated using Teichholtz and Troy formulas, respectively. The ejection fraction was determined to assess systolic function. Generally, at least 3 stable cardiac cycles were averaged to obtain the reported parameters.

#### 4.3.3 Western‐blot analysis of autophagy and senescence markers

Left ventricles were collected at sacrifice and cut into three parts longitudinally before being snap frozen in liquid nitrogen. One‐third was used for protein extraction, with mechanical disruption performed in RIPA buffer (Bertin Instruments, #P000972‐LYSK0‐A.0). Heart lysates were denatured for 5 min at 100°C in LDS‐reducing buffer (Invitrogen, #NP0008 and #NP0009). Immunoblotting was performed following standard protocols (Montégut et al., 2022). Briefly, proteins were separated by SDS‐PAGE on 4%–12% bis–tris polyacrylamide gels and transferred to 0.22 μm nitrocellulose membranes. After blocking, the membranes were incubated overnight at 4°C with the following primary antibodies and dilutions: anti‐DBI (Abcam, #231910, 1:1000), anti‐p62 (Abnova, #H00008878), anti‐LC3B (Cell Signaling, #2775), anti‐p16 (Abcam, #ab211542) and anti‐GAPDH (Cell Signaling, #2118). Species‐specific HRP‐conjugated secondary antibodies (SouthernBiotech, #1031‐05 or #4049‐05, 1:5000) were incubated for 1 h at room temperature before chemiluminescent revelation and imaging. Densitometric quantifications were performed with ImageQuantTL (version 8.1, GE Healthcare) and protein contents were normalized to the housekeeping protein GAPDH density on each lane.

### 4.4 Statistical analyses

Continuous variables were described by the mean and standard deviation or SEM and categorical variables as numbers and percentages. ACBP/DBI plasma levels at baseline in patients with future cancer, cardiovascular disease, cancer or cardiovascular disease and controls were represented as box and whisker plots (mean, first, and third quartiles) and compared using one‐tailed unpaired *t*‐tests to test the hypothesis that ACBP/DBI levels would be higher in patients with future age‐related diseases.

To account for the differences between these groups in terms of age and BMI, patients were matched with three (cancer and cardiovascular disease) or two (cancer or cardiovascular disease) controls with similar age and BMI, and ACBP levels between patients and their matched controls were compared using one‐tailed unpaired *t*‐tests.

The relation between DBI and other continuous parameters is represented as dots with one or more regression lines according to subgroups. Pearson's correlation coefficients with their p values were calculated. The number of samples available is featured on each plot. Multivariate analysis was performed with a generalized linear model to test the independence of correlation between ACBP/DBI and the other variables with age, and logistic regression when testing the association of continuous variables with cancer or CVD occurrence.

To have an estimate of the correlation coefficient across different cohorts with and without cancer, we did a meta‐analysis of available cohorts (Bravo‐San Pedro, Sica, Martins, Anagnostopoulos, et al., 2019; Joseph et al., 2021), using Fisher's z transformation of correlations. Pearson's correlation coefficients from each study and the pooled estimate are represented with their 95% confidence interval in forest plots. Heterogeneity was assessed using the *I* ^2^ statistic. Because of the expected heterogeneity of the cohorts, all meta‐analyses were performed using a conservative random‐effects model.

A *p*‐value below 0.05 was considered significant. Statistics were managed using R software version 3.6.0 (R Foundation for Statistical Computing; https://www.R‐project.org/).

For the *S. cerevisiae* experiments, quantitative data represent means ± SEM of at least three independent experiments/clones, as detailed in each figure legend. Statistical analyses were performed using Student's *t*‐test (one‐tailed, unpaired) for heat stress and densitometric analysis, or 2‐way ANOVA for chronological aging experiments.

## AUTHOR CONTRIBUTIONS

LM performed data analyses (Figures 2, 3, 4, 5, Figures S3–S8) and contributed to the cardiac experiments, especially at the levels of immunoblots (Figure 6). AJ performed data analyses (Figures 2, 3, 4, 5, Figures S3–S8, Tables S1 and S2). HC and IM optimized the ACBP/DBI ELISA and performed the measurements of circulating ACBP/DBI (Figure S2, Figures 2, 3, 4, 5, Figures S3–S8). MA performed the cardiac experiments and their analyses (Figure 6). CR, SD, and FM performed and interpreted the yeast experiments (Figure 1, Figure S1). OM, FV, GA, and SL contributed to mouse experiments (Figure 6). AL, FG, BB, and FF provided human plasma samples and clinical information with respect to the cohorts. IM, FM, and GK wrote the paper. GK conceived the study. All authors revised and approved the paper.

## CONFLICTs OF INTEREST

GK has been holding research contracts with Daiichi Sankyo, Eleor, Kaleido, Lytix Pharma, PharmaMar, Osasuna Therapeutics, Samsara Therapeutics, Sanofi, Sotio, Tollys, Vascage, and Vasculox/Tioma. GK has been consulting for Reithera. GK is on the Board of Directors of the Bristol Myers Squibb Foundation France. GK is a scientific cofounder of everImmune, Osasuna Therapeutics, Samsara Therapeutics, and Therafast Bio. GK is the inventor of patents covering therapeutic targeting of aging, cancer, cystic fibrosis, and metabolic disorders. FM has an equity interest in and is an advisor of TLL The Longevity Labs GmbH and has an equity interest in Samsara Therapeutics.

## Supporting information


FigureS1‐S9

Table S1

Table S2

## Data Availability

Immunoblot results regarding Figure 1 and Figure 6 are openly available at Mendeley data (https://data.mendeley.com/datasets/sh7gf8jft5). The clinical data that support the findings of this study (Figures 2, 3, 4, 5, Figures S3–S8, Tables S1 and S2) are available on request from the corresponding author. These data are not publicly available due to privacy or ethical restrictions. All other data that support the findings of this study (Figure 1, Figure S1, and Figure 6) are available from the corresponding author upon reasonable request.
