# Supplementary material for: High plasma concentrations of acyl‐coenzyme A binding protein (ACBP) predispose to cardiovascular disease: Evidence for a phylogenetically conserved proaging function of ACBP
Source: Aging Cell. 2022 Dec 12;22(1):e13751. doi: 10.1111/acel.13751 (PMC9835587; doi:10.1111/acel.13751)
Supplement: Supplementary file 1 — FigureS1‐S9 [file ACEL-22-e13751-s001.pdf]

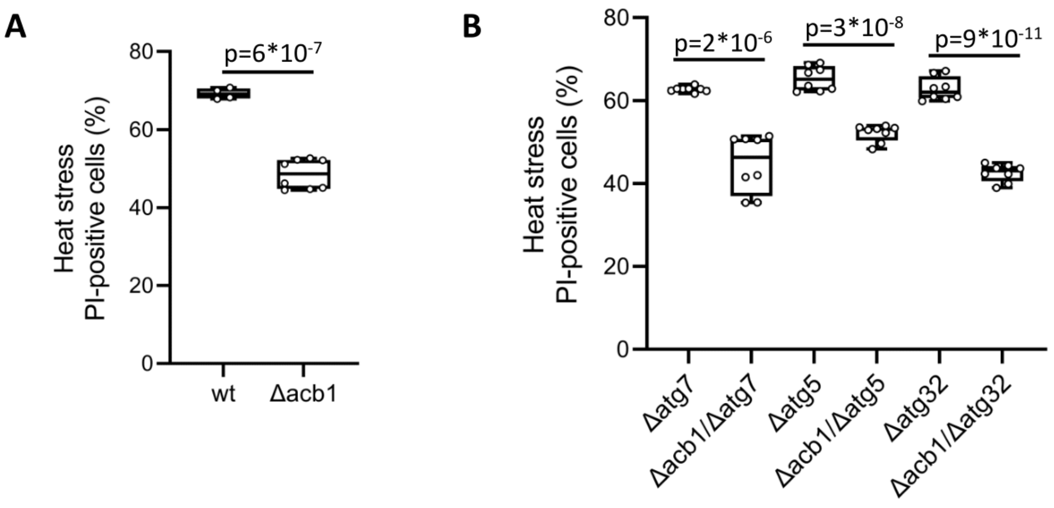

Suppl. Fig. 1: Acb1 - deficient *Saccharomyces cerevisiae* shows enhanced heat stress resistance

(**A**) Logarithmic growing yeast strains (wt and Δ*acb1*) were submitted to heat stress (50°C) and dead cells were identified via flow cytometry analysis following propidium iodide (Pi) staining.
(**B**) Autophagy incompetent yeast Acb1 - deficient diploid *S. cerevisiae* mutants (Δ*acb1*/Δ*atg7*, Δ*acb1*/Δ*atg5*, Δ*acb1*/Δ*atg*32) still showed enhanced heat stress resistance when compared to respective autophagy incompetent single gene deletion mutants.
Results are reported as means ± SEM (n = 4 to 8). Statistical analyses were performed using unpaired one-sided Student's t-test, the resulting p values are presented on the graphs.

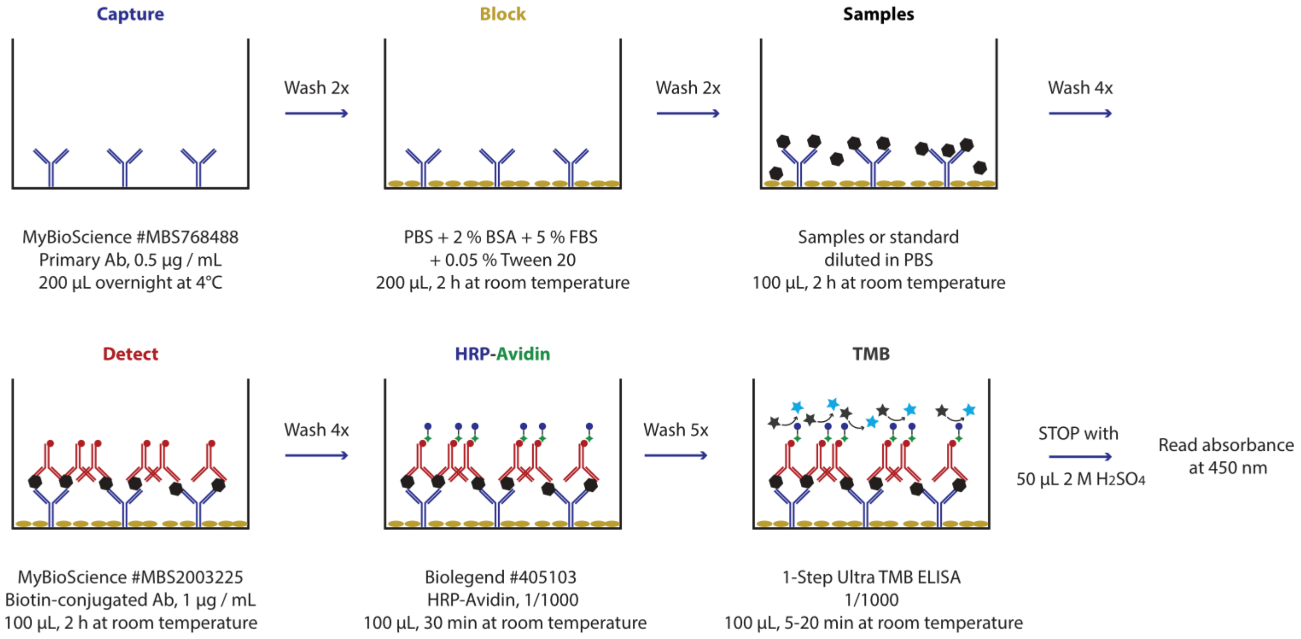

Suppl. Fig. 2: Schematic protocol of homemade ELISA

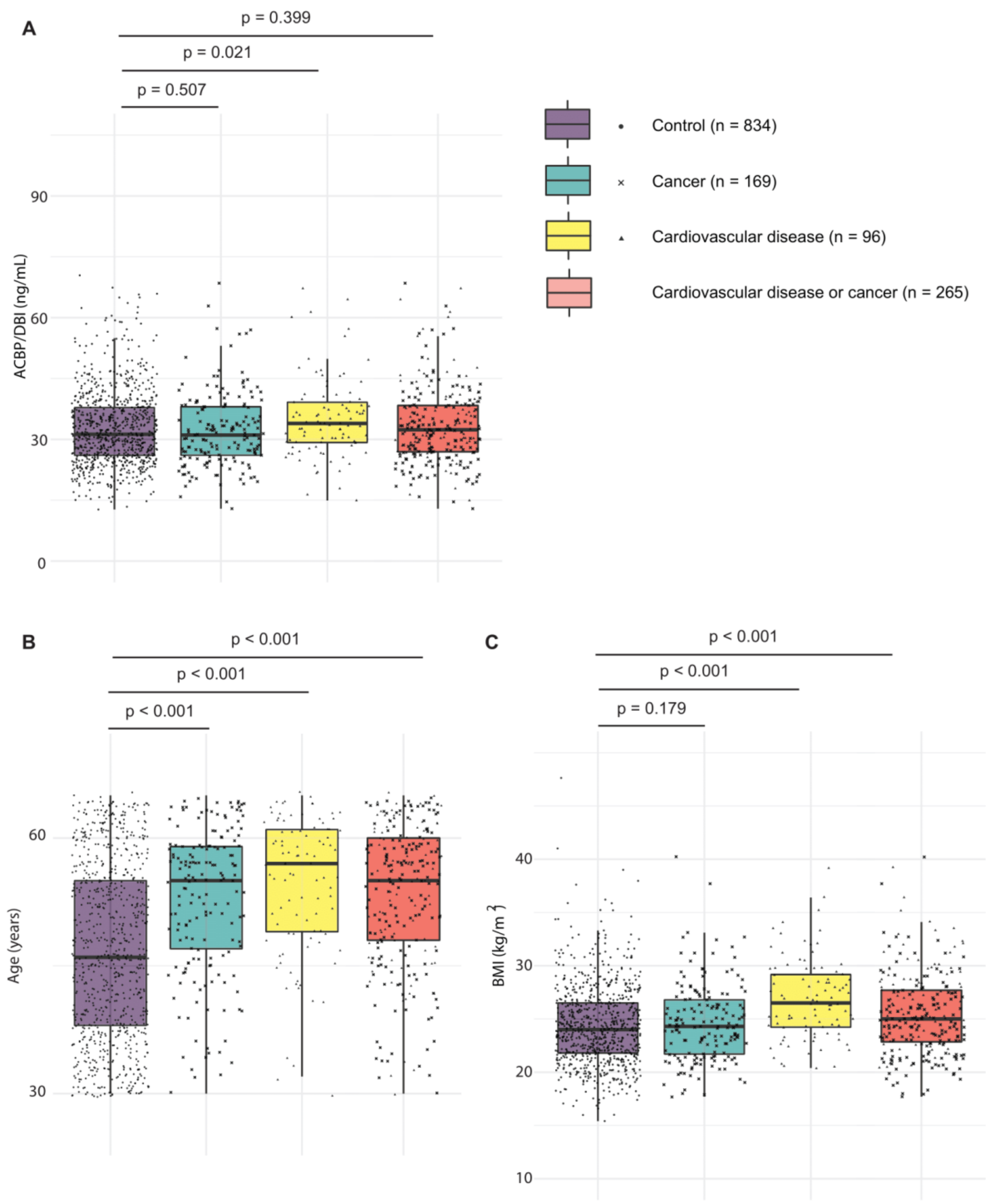

Suppl. Fig. 3: Comparison between controls, cancer alone, CVD alone and cancer or cardiovascular disease for age, BMI and ACBP/DBI

Box and whisker plots representing age (years) (**A**), body mass index (kg/m^2) (**B**) and ACBP/DBI levels (ng/mL) (**C**) in patients who later developed a cancer, a cardiovascular disease, either a cancer or a cardiovascular disease or neither of these complications (controls) from the DESIR 2 cohort. Statistical analyses were performed using one-sided Student's t-test, the resulting p values are presented on the graphs.

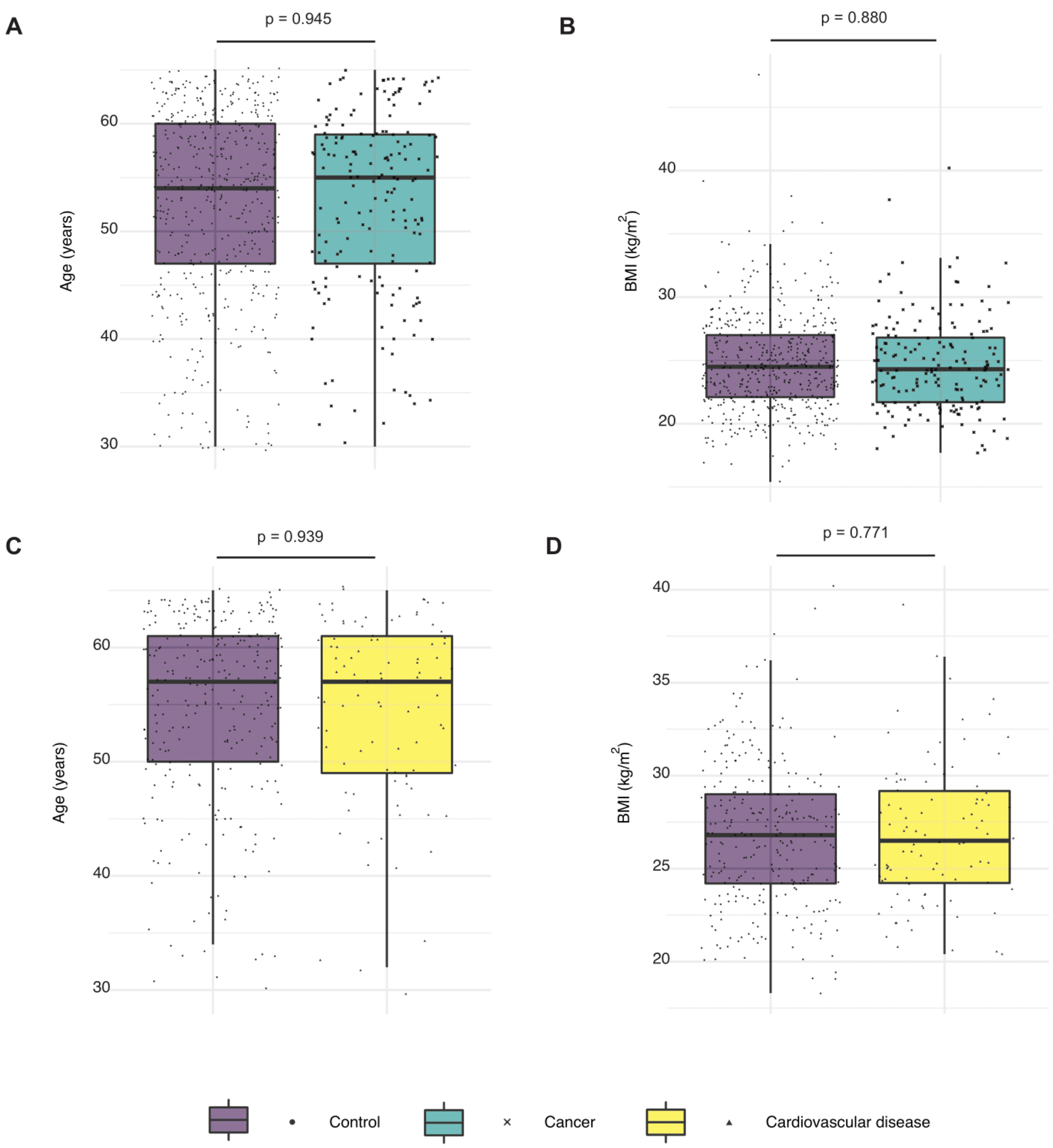

Suppl. Fig. 4: Comparison of age or BMI levels between cancer and cardiovascular disease patients and controls matched for age and BMI

Box and whisker plots representing age (**A**, **C**) or BMI (**B**, **D**) levels between cancer (**A**, **B**) and cardiovascular disease (**C**, **D**) patients and their matched controls from the DESIR 2 cohort. Statistical analyses were performed using two-sided Student's t-test, the resulting *p* values are presented on the graphs.

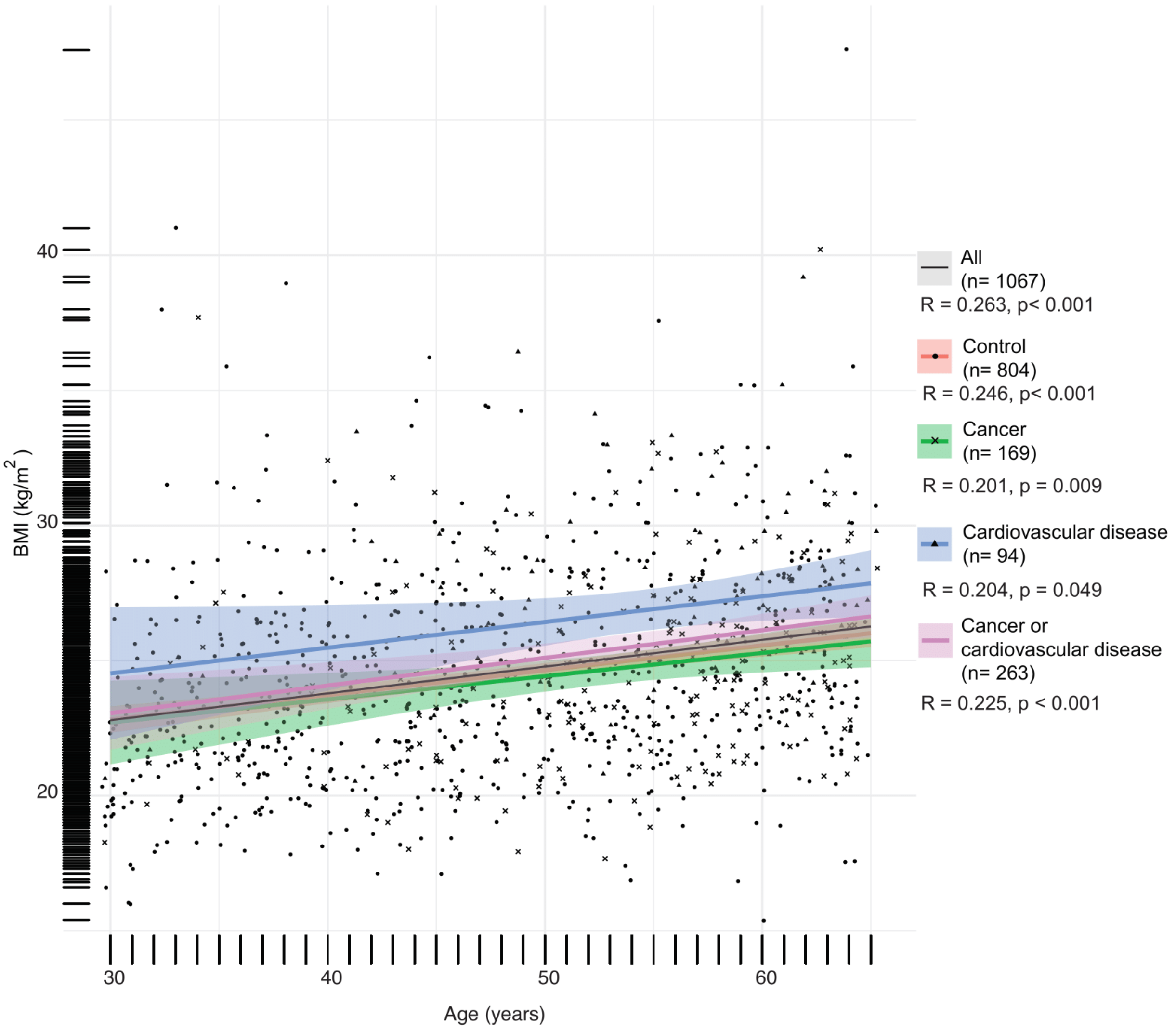

Suppl. Fig. 5: Correlation between age and BMI for all patients, controls, cancer alone, cardiovascular disease alone and cancer or cardiovascular disease

Scatter plot with regression line between body mass index (kg/m^2) and age in patients who later developed a cancer, a cardiovascular disease, either a cancer or a cardiovascular disease or neither of these complications (controls) from the DESIR 2 cohort. Pearson's correlation coefficient (R) with their p value and the number of samples available (n) are shown in the legend of each panel.

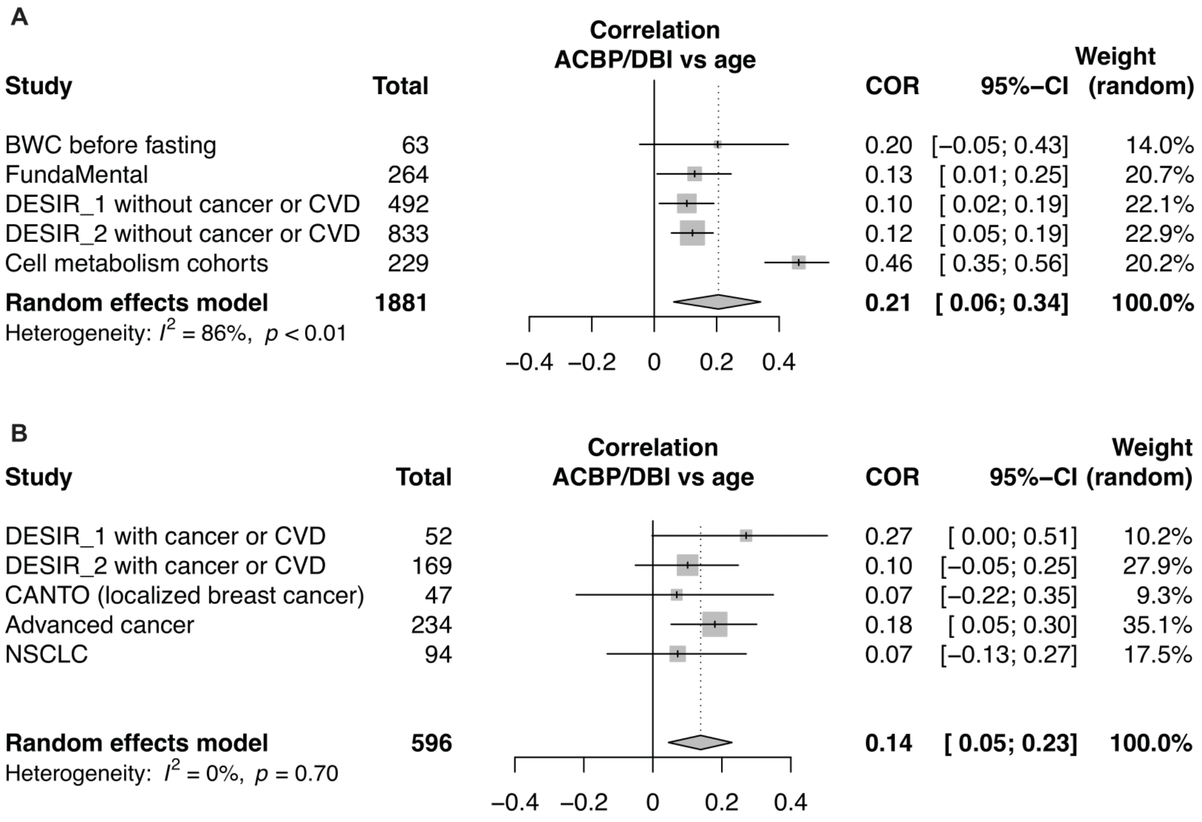

Suppl. Fig. 6: Meta-analysis of the correlation between ACBP/DBI and age in patients without and with cancer

Aggregated data from patients without (**A**) and with (**B**) cancer from this study and previously published cohorts were combined to calculate the pooled correlation by random effect model. Each Pearson's correlation coefficient is represented with its 95% confidence interval. The size of the square is proportional to the sample size of the study.

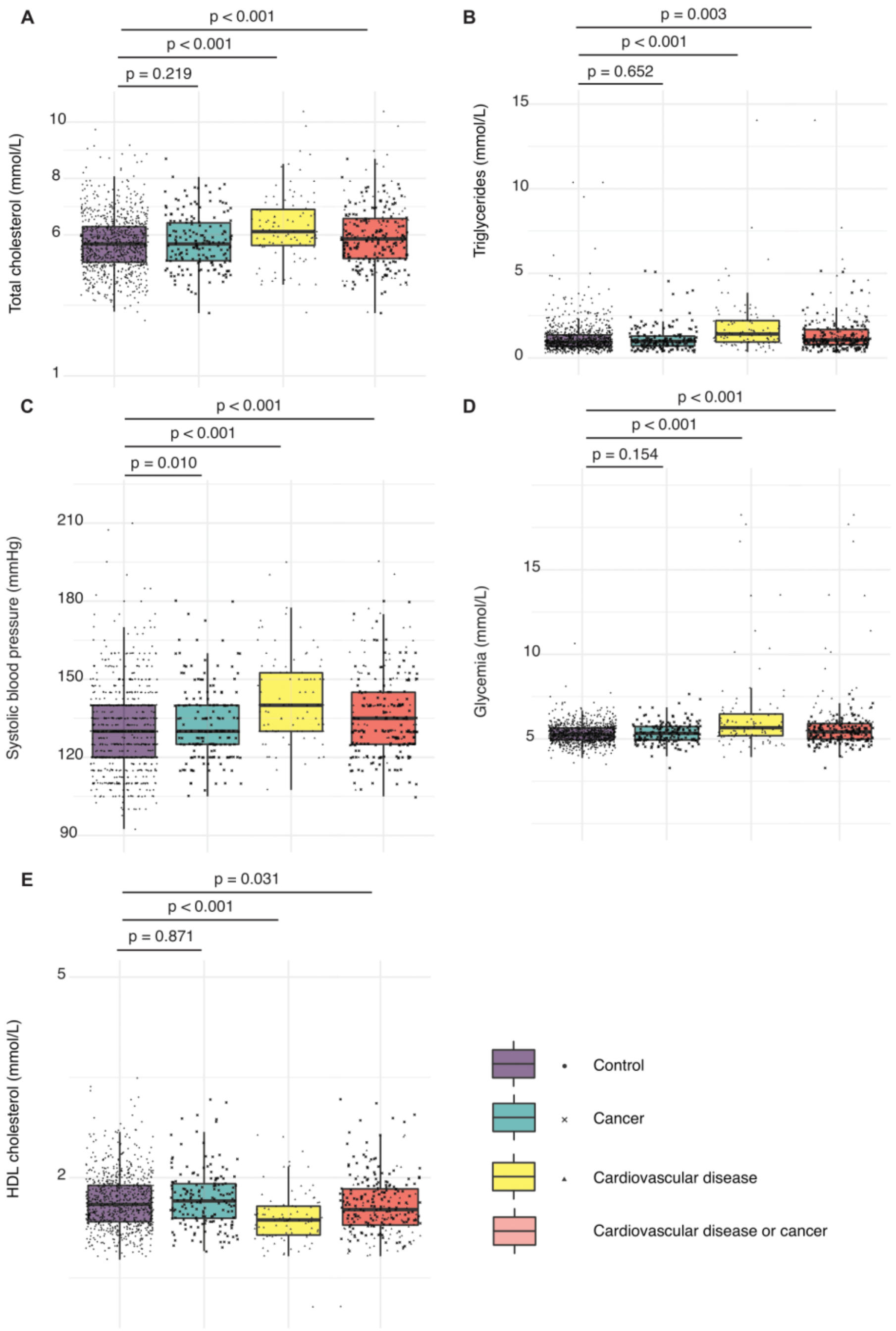

Suppl. Fig. 7: Comparison between controls, cancer alone, CVD alone and cancer or cardiovascular disease for total cholesterol, triglycerides, systolic blood pressure, glycemia and HDL cholesterol

Box and whisker plots representing total cholesterol (mmol/L) (**A**) triglycerides (mmol/L) (**B**), systolic blood pressure (mmHg) (**C**), glycemia (mmol/L) (**D**) and HDL cholesterol (**E**) in patients who later developed cancer, cardiovascular disease, either cancer or cardiovascular disease or neither of these complications (controls) from the DESIR 2 cohort. Statistical analyses were performed using one-sided Student's t-test, the resulting p values are presented on the graphs.

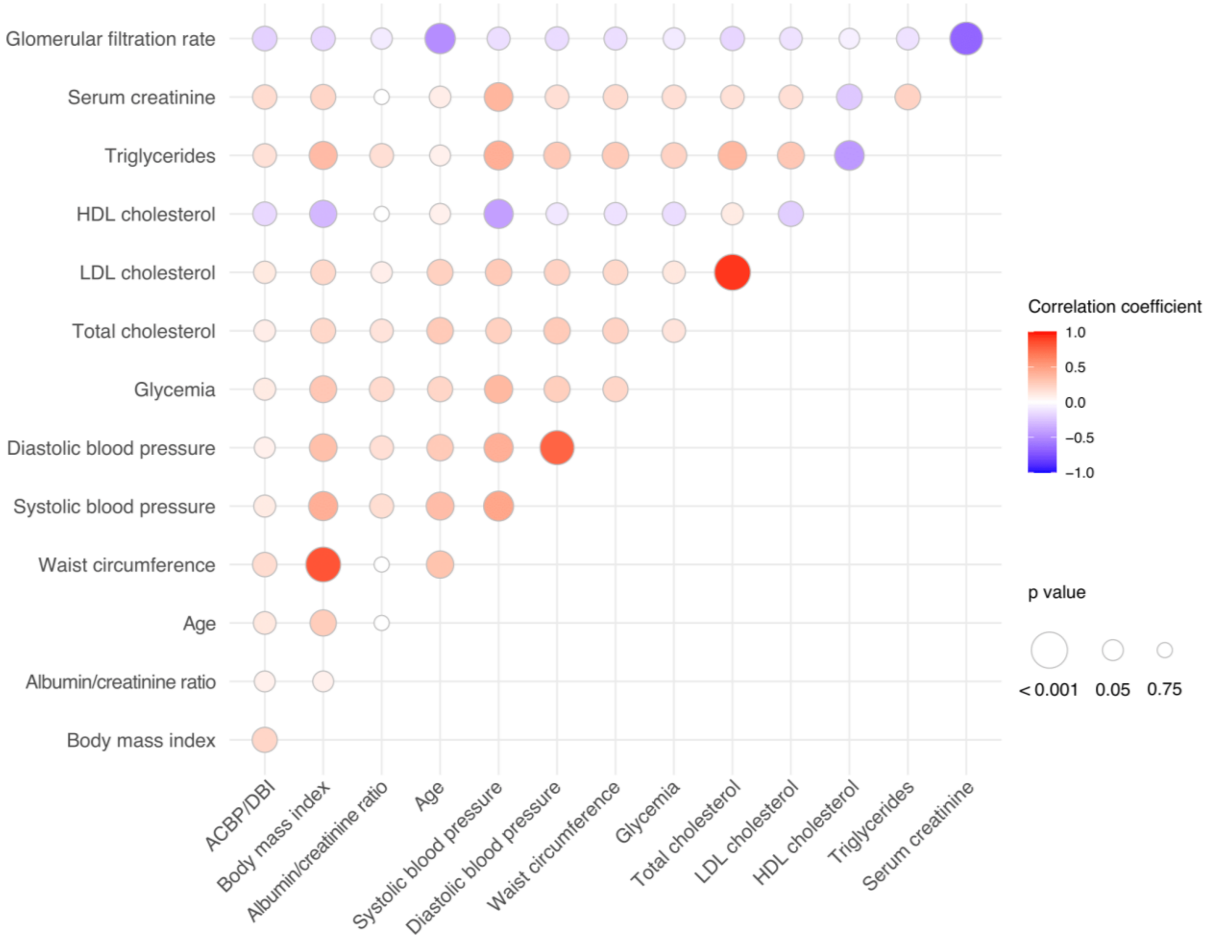

Suppl. Fig. 8: Correlation plot between biological and anthropometric characteristics of DESIR 2 patients

Correlation matrix representing the correlations between ACBP/DBI, body mass index, albumin creatinine ratio, age, albumin/creatinine ratio, waist circumference, systolic and diastolic blood pressure, glycemia, total, LDL and HDL cholesterol, triglycerides, serum creatinine and glomerular filtration rate. The Pearson's correlation coefficient (R) is represented by the intensity of the color of the circle (with red indicating a negative correlation and blue a positive correlation), whereas the statistical significance is represented by the size of the circle.

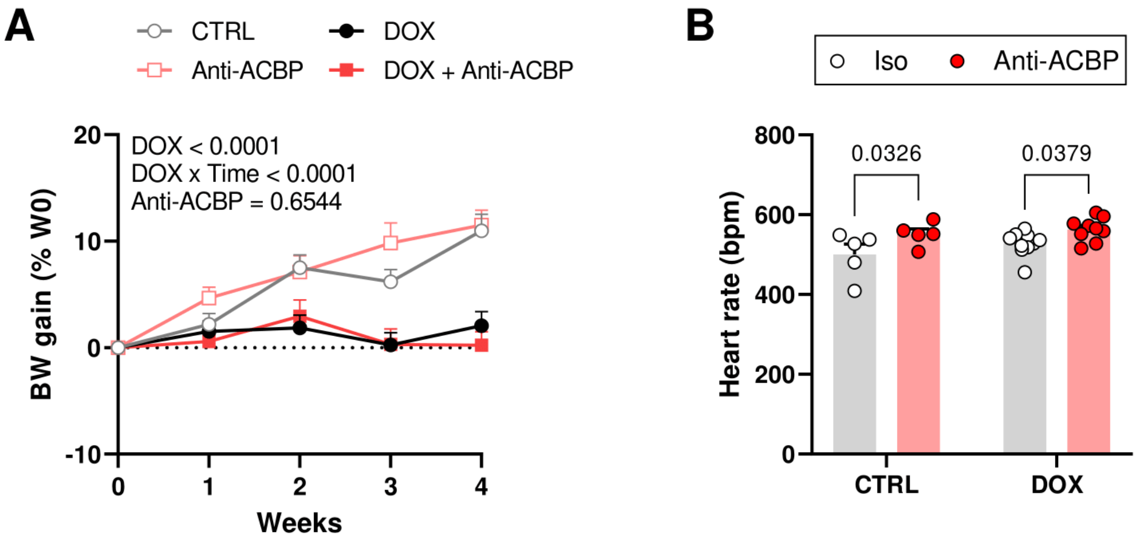

Suppl. Fig. 9. Anti-ACBP effects on body weight and heart rate in doxorubicin-treated mice

(**A**) Body weight (BW) gain and (**B**) heart rate in C57Bl/6J female mice that were treated with an ACBP-neutralizing antibody (Anti-ACBP) or mouse isotype IgG (CTRL) upon anthracycline-induced cardiotoxicity using doxorubicin (DOX).

N=5-10 mice per groups. P values in (A) were calculated using using a mixed model, including DOX, anti-ACBP and Time as fixed factors. In (B), P values represent pairwise comparisons between anti-ACBP-treated mice and their respective isotype (Iso)-treated controls using simple main effects of a factorial ANOVA. Bars and error bars show means and SEM, respectively, with individual data points superimposed. Abbreviations: bpm, beat per minute.
